# Barttin Regulates the Subcellular Localization and Posttranslational Modification of Human Cl^-^/H^+^ Antiporter ClC-5

**DOI:** 10.3389/fphys.2018.01490

**Published:** 2018-10-23

**Authors:** Daniel Wojciechowski, Elena Kovalchuk, Lan Yu, Hua Tan, Christoph Fahlke, Gabriel Stölting, Alexi K. Alekov

**Affiliations:** ^1^Institute for Neurophysiology, Hannover Medical School, Hanover, Germany; ^2^Institute of Complex Systems 4 (ICS-4) – Zelluläre Biophysik, Forschungszentrum Jülich, Jülich, Germany

**Keywords:** ClC-5, barttin, CLC transport, kidney, Dent disease, Bartter syndrome, Golgi bypass

## Abstract

Dent disease 1 (DD1) is a renal salt-wasting tubulopathy associated with mutations in the Cl^-^/H^+^ antiporter ClC-5. The disease typically manifests with proteinuria, hypercalciuria, nephrocalcinosis, and nephrolithiasis but is characterized by large phenotypic variability of no clear origin. Several DD1 cases have been reported lately with additional atypical hypokalemic metabolic alkalosis and hyperaldosteronism, symptoms usually associated with another renal disease termed Bartter syndrome (BS). Expression of the Bartter-like DD1 mutant ClC-5 G261E in HEK293T cells showed that it is retained in the ER and lacks the complex glycosylation typical for ClC-5 WT. Accordingly, the mutant abolished CLC ionic transport. Such phenotype is not unusual and is often observed also in DD1 ClC-5 mutants not associated with Bartter like phenotype. We noticed, therefore, that one type of BS is associated with mutations in the protein barttin that serves as an accessory subunit regulating the function and subcellular localization of ClC-K channels. The overlapping symptomatology of DD1 and BS, together with the homology between the proteins of the CLC family, led us to investigate whether barttin might also regulate ClC-5 transport. In HEK293T cells, we found that barttin cotransfection impairs the complex glycosylation and arrests ClC-5 in the endoplasmic reticulum. As barttin and ClC-5 are both expressed in the thin and thick ascending limbs of the Henle’s loop and the collecting duct, interactions between the two proteins could potentially contribute to the phenotypic variability of DD1. Pathologic barttin mutants differentially regulated trafficking and processing of ClC-5, suggesting that the interaction between the two proteins might be relevant also for the pathophysiology of BS. Our findings show that barttin regulates the subcellular localization not only of kidney ClC-K channels but also of the ClC-5 transporter, and suggest that ClC-5 might potentially play a role not only in kidney proximal tubules but also in tubular kidney segments expressing barttin. In addition, they demonstrate that the spectrum of clinical, genetic and molecular pathophysiology investigation of DD1 should be extended.

## Introduction

The X-linked hypercalciuric nephrolithiasis Dent disease 1 (DD1, OMIM#300009) is linked to mutations in the *CLCN5* gene. The encoded protein ClC-5 is a Cl^-^/H^+^ antiporter (Picollo and Pusch, 2005; Scheel et al., 2005). It has been established that its malfunction in DD1 affects mainly the kidney proximal tubules (PT) by altering vesicular chloride homeostasis, impairing endocytosis and reducing endosomal acidity and acidification rate (Piwon et al., 2000; Günther et al., 2003; Hara-Chikuma et al., 2005; Novarino et al., 2010). In addition, ClC-5 regulates the trafficking and processing of the megalin/cubilin endocytic receptor complex, and of several ion transporters like the sodium/proton exchanger Nhe3, the sodium/phosphate cotransporter Npt2a, and kidney ATP-dependent H^+^-pumps (Piwon et al., 2000; Christensen et al., 2003; Moulin et al., 2003; Lin et al., 2011).

The clinical manifestations of DD1 that ultimately lead to progressive renal failure include urolithiasis, nephrocalcinosis, urinary loss of low molecular weight proteins, glucose, amino acids, phosphate and calcium (Dent and Friedman, 1964; Wrong et al., 1994; Lloyd et al., 1996; Scheinman, 1998). Two recent reports show that mild progressive hypokalemia is also common in DD1 (Mansour-Hendili et al., 2015; Blanchard et al., 2016). Despite its monogenic origin, DD1 is characterized by a profound phenotypic variability with no clear origin (see recent summary in Mansour-Hendili et al., 2015). The two currently available ClC-5 knock-out mouse models also exhibit significant differences, especially in regard to calcium metabolism (Piwon et al., 2000; Wang et al., 2000). This particular difference has led to the notion that other proteins might also play a role in the disease pathophysiology (Devuyst and Guggino, 2002; Günther et al., 2003). Yet, no candidate for such a protein has been identified up to now.

**FIGURE 1:**
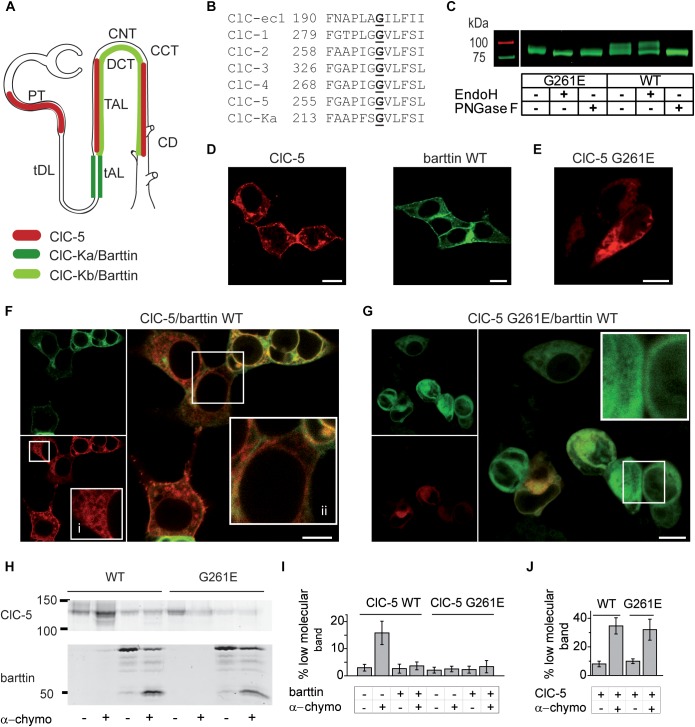
Interaction between ClC-5 and barttin in non-polarizing HEK293T cells. **(A)** Major expression sites of ClC-5 and ClC-K/barttin reported in the literature (PT, proximal tubule; tDL, thin descending limb of Henle’s loop; tAL and TAL, thin and thick ascending limbs of the Henle’s loop; DCT, distal convoluted tubule; CNT, connecting tubule; CCT, cortical collecting tubule; CD, collecting duct). **(B)** Alignment showing the sequence conservation of the protein region containing ClC-5 G261E, a Dent disease 1 mutation with Bartter-like phenotype (bold). **(C)** False-color representation of a fluorescent SDS-PAGE gel of HEK293T cell lysates containing expressed ClC-5-mYFP WT or ClC-5-mYFP G261E. Lysates were incubated with PNGaseF or EndoH to cleave all types or specifically the high mannose N-linked glycosylation, respectively. The resistance of ClC-5 to EndoH indicates complex glycosylation. **(D)** Representative confocal images of HEK293T cells expressing ClC-5 mCherry or barttin mCFP. Scale bars here and hereafter correspond to 10 μm. **(E)** Representative confocal image of HEK293T cells expressing ClC-5 G261E mCherry. **(F,G)** Representative confocal images of HEK293T cells coexpressing barttin (green) together with ClC-5 WT or ClC-5 G261E (ClC-5 in red). Magnified regions of interest are included as insets [in panel **(F)**, “i” denotes ER staining, whereas “ii” denotes staining of the perinuclear space]. **(H)** Grayscale presentation of a fluorescent SDS-PAGE gel of HEK293T cell lysates with expressed ClC-5-mCerulean or ClC-5-mCerulean G261E with or without coexpressed barttin mCherry. A brief exposure of intact cells to α-chymotrypsin was used to selectively cleave surface-exposed proteins. **(I)** Percentage of the low molecular ClC-5 protein band obtained from densitometry analysis of data as depicted in panel **(H)**, *n* = 7–11. The intensity of the lower band increases due to cleavage of surface exposed proteins by α-chymotrypsin and is proportional to the PM abundance of the investigated protein. **(J)** Percentage of the low molecular barttin protein band obtained from densitometry analysis of data as depicted in panel **(H)**, *n* = 7–11.

In the last years, several atypical monogenic DD1 cases have been reported, in which patients carrying ClC-5 mutations (Supplementary Table 1) additionally exhibit symptoms like normochloremic hypokalemic metabolic alkalosis and/or growth hormone deficiency (Besbas et al., 2005; Sheffer-Babila et al., 2008; Bogdanović et al., 2010; Okamoto et al., 2012). To our knowledge, the effects of these mutations on ClC-5 function have not been investigated. The atypical symptoms are usually not observed in DD1 but are characteristic for Bartter syndrome (BS), another hereditary renal disease (Bartter et al., 1962). The overlapping disease symptomatology prompted us to assume that proteins implicated in BS might also regulate ClC-5 transport. Our attention was specifically drawn by the protein barttin that is associated with Bartter syndrome 4a (BS4a, OMIM#602522). Barttin does not have a transport function but acts as accessory subunit regulating the function and localization of ClC-K type chloride channels that belong to the same protein family as ClC-5 (Birkenhäger et al., 2001; Estévez et al., 2001). Based on the homology between proteins of the CLC family, we hypothesized that barttin might be also able to regulate the function and localization of ClC-5. Remarkably, expression of ClC-5 has been detected not only in the PT, which is devoid of barttin, but also in kidney segments with significant barttin expression such as the thick ascending limb of the Henle’s loop (TAL), and in intercalated cells of the collecting duct (ICCD) (Günther et al., 1998; Luyckx et al., 1998; Devuyst, 1999; Sakamoto et al., 1999; Nanami et al., 2015; Ogawa et al., 2017;Figure 1A).

Of note, no mutations in genes encoding for proteins linked to Bartter-like symptoms (specifically, NCCT, ClC-Kb, Kir1.1, and NKCC2) have been discovered in the aforementioned atypical DD1 cases but the existence of barttin mutations has not been investigated (Besbas et al., 2005; Bogdanović et al., 2010; Okamoto et al., 2012). To test whether barttin can regulate ClC-5 function, we cotransfected wild type (WT) and various pathogenic mutants of barttin and ClC-5 in non-polarizing mammalian HEK293T (human embryonic kidney) cells and explored the consequences of this maneuver by biochemistry, confocal microscopy, and electrophysiology.

## Materials and Methods

### Mutagenesis and Heterologous Expression

Mutation G261E was introduced into existing ClC-5-mCherry, ClC-5-mYFP and ClC-5-mCerulean fusion proteins (Alekov, 2015), all in the pRcCMV expression vector, using the QuikChange site-directed mutagenesis kit (Agilent Technologies). The expression vector p156rrL with ClC-5 GFP was kindly provided by Dr. R Guzman (Institute of Complex Systems 4 (ICS-4) – Zelluläre Biophysik, Forschungszentrum Jülich, D-52425 Jülich, Germany). Existing pcDNA3.1 (+) expression vectors encoding the fusion proteins barttin-mCFP or barttin-mCherry mutants were used to express barttin. For electrophysiology and biochemical analyses, 10 μg of ClC-5-encoding plasmids were transiently transfected in HEK293T cells grown in 10-cm Petri dishes (Sarstedt), alone or with 5 μg (unless otherwise indicated) barttin-encoding plasmids using Lipofectamine 2000 (Thermo Fisher) or calcium phosphate precipitation. Electrophysiological recordings and biochemical analysis were performed 24–48 h, confocal imaging – 24 h after transfection, respectively.

### Confocal Microscopy

Live cell confocal imaging was performed on a Zeiss LSM 780 AxioObserver microscope (Zeiss) with C-Apochromat 40×/1.20 water immersion objective. Barttin-mCFP fusion proteins were excited at 440 nm, emission was detected at 455–580 nm. ClC-5-mCherry fusion proteins were excited at 561 nm, emission was detected at 580–670 nm. For colocalization analysis, ClC-5-mYFP was excited at 514 nm and imaged at 520–560 nm. ER-Tracker Red (Thermo Fisher Scientific) was applied to these cells according to the manufacturer’s manual to stain the cell endoplasmic reticulum and imaged using the mCherry microscope settings.

### Protein Biochemistry

PNGaseF and EndoH (New England Biolabs) were used to determine the type of the ClC-5 glycosylation. The supplier’s protocol was adapted for shorter denaturation to preserve the functionality of the fluorescence tags. The analysis was performed by fluorescence scanning on a Typhoon FLA 9500 (GE Healthcare). The mYFP tag was excited at 473 nm and its fluorescence recorded using a 530/20 bandpass filter. Protein size was estimated using a standardized marker (Precision Plus Protein Dual Color Standards, BioRad). To quantify the effect of barttin on the glycosylation of ClC-5, transiently transfected HEK293T cells were lysed in buffer containing: NaCl (150 mM), HEPES (10 mM), Triton X-100 (1%) supplemented with protease inhibitor mix (1%, Roche complete) at pH 7.4. Lysates were cleared by a centrifugation step (13000 rpm, 15 min, 4 °C). Proteins were separated by 12% SDS-PAGE and imaged using fluorescence scanner (Fusion SL, BioRad). mCherry and mYFP ClC-5 fusions were excited with a LED at 505–550 nm. Fluorescence emission was detected with a 620/52 (or 565/20) bandpass filter. Barttin-mCFP fluorescence was elicited with a LED illumination at 360–480 nm and its emission was detected with a 542/25M bandpass filter.

Exposure of intact cells expressing ClC-5 and/or barttin to α-chymotrypsin (Sigma) was used to estimate the PM abundance of the proteins. In particular, 48 h after transfection, HEK293T cells were washed three times with 3 ml PBS, before adding 2 ml of PBS with or without 0.3 μg/ml chymotrypsin. After a 7-min incubation at 37°C, chymotrypsin was inactivated by freshly prepared 0.5 mM PMSF solution (Phenylmethylsulfonyl fluoride, Sigma). Subsequently, cells were transferred into Eppendorf tubes, washed by three repetitive centrifugations (each 1 min, 2400 rpm), and resuspended in 500 μl PBS. Cells were lysed in 250 μl lysis buffer (see above) for 30 min at 37°C under continuous shaking. 50 μl of cleared lysates were subjected to SDS-PAGE analysis as described above.

Membrane proteins for immunoprecipitation were solubilized from 80%-confluent 10-cm Petri dishes of HEK293T cells transfected with barttin-mCherry together with or without ClC-5-mVenus using ComplexioLyte 47a (Logopharm). 400 μl of the cleared lysate were incubated for 1h with 1 μg of monoclonal anti-GFP antibody (Life Technologies) (described in more detail in Stölting et al., 2015). The same amount of cleared lysate was used as a control for unspecific binding and processed further without antibody. Antibody-bound proteins were purified by 2-h incubation with protein G-sepharose beads (Thermo Fisher Scientific) and eluted using 50 μl of 2× SDS loading buffer. Samples were run on a 10% SDS gel and analyzed after fluorescence scanning on a Typhoon FLA 9500 (GE Healthcare). mVenus was excited at 473 nm and its fluorescence recorded using a 530/20 bandpass filter. The signal of mCherry was recorded using a 532 nm laser and a long pass 575 nm filter.

### Electrophysiology

Whole-cell patch-clamp recordings were performed using either an Axopatch 200B (Molecular Devices, Sunnyvale, CA, United States) or EPC10 (HEKA Electronics, Germany). Borosilicate pipettes (ALA Scientific) with resistances of 1–2 MΩ were pulled on an automated puller (Sutter) and fire-polished. Capacitive cancellation and series resistance compensation were applied to reduce capacitive artifacts and series resistance errors, resulting in voltage errors not exceeding 5 mV. Currents were digitized at 50 kHz sampling rate after analog filtering at 3–10 kHz with a low-pass Bessel filter. The standard extracellular solution contained (in mM): NaCl 145, Hepes 15, KCl 4, CaCl_2_ 2, MgCl_2_ 1, pH 7.4. The standard intracellular solution contained (in mM): NaCl 105, Hepes 15, MgCl_2_ 2, EGTA 5, pH 7.4. P/4 leak subtraction was performed by applying repeating voltage steps with a -60-mV baseline to minimize capacitance artifacts. For electrophysiological characterization of the effects of barttin on ClC-5 function, only cells with higher mCFP (barttin) than YFP (ClC-5) fluorescence intensity were used.

### Simultaneous Fluorescence and Current Recordings

Experiments correlating currents with the amount of expressed ClC-5-mCerulean fusion proteins were conducted similarly as described previously (Ronstedt et al., 2015). In brief, transfected HEK293T cells were cultivated in 3-cm IBIDI dishes and mounted on an inverted IX71 microscope with UPlanSApo 60X/1.35 oil immersion objective (both from Olympus). mCerulean was excited at 440 nm using a Polychrome V monochromator; emitted fluorescence was detected at 490 nm using a photodiode equipped ViewFinder III (Till Photonics). Fluorescence values were measured in the linear range of the photodiode detector and are given as arbitrary units (a.u.). Background fluorescence values and current amplitudes were measured on untransfected cells and found to be negligible. The coexpression of barttin-mCherry was controlled by excitation at 560 nm and observation at 610 nm. Steady-state ClC-5 current amplitudes at +145 mV were plotted versus the corresponding fluorescence values measured in the same cells. The so obtained plots were fitted with standard linear functions.

### Data Analysis and Statistics

Data analysis and visualization were performed using a combination of pClamp (Molecular Devices), FitMaster (HEKA), Excel (Microsoft), and SigmaPlot (Jandel Scientific). Confocal images were assembled for publication using ImageJ (Rasband, W.S., ImageJ, United States NIH, Bethesda, MD, United States). Colocalization analysis was performed after spectral unmixing using the JACoP plugin of ImageJ. Details on experiment and cell numbers are provided in the figure legends and the main text. Statistical analyses were performed using Student’s *t*-test. Normal value distribution was assumed. All data are presented as mean ± SEM.

## Results

### The Bartter-Like DD1 Mutant G261E Exhibits Defective N-Glycosylation and Is Retained in the ER

Among the published mutations associated with Bartter-like DD1, we selected the single amino acid exchange G261E (Figure 1B) that (to our knowledge) is the only mutation expected to encode a non-truncated protein with the size of WT ClC-5 (Besbas et al., 2005; Sheffer-Babila et al., 2008; Bogdanović et al., 2010; Okamoto et al., 2012; see Supplementary Table 1). Typically for DD1, the proteinuria and hypercalciuria were diagnosed in the affected patient; however, atypical hypokalemic metabolic alkalosis, hyperreninemic hyperaldosteronism, and growth failure associated with partial growth hormone deficiency were also present (Bogdanović et al., 2010). No mutations in proteins associated with barter-like symptoms were found in the patient (the Na/K/Cl cotransporter NKCC2, the potassium channel Kir 1.1a, the Na/Cl cotransporter NCCT, and the chloride channel ClC-Kb) but the existence of mutations or polymorphisms in barttin was not investigated (Bogdanović et al., 2010).

ClC-Kb Biochemical analysis showed that ClC-5 G261E is expressed at full length and revealed a defect in the processing of the mutant (Figure 1C). In accordance with the literature (Jouret et al., 2004), a significant percentage of ClC-5 WT was complexly glycosylated. In contrast, the complex glycosylation of the mutant was impaired (Figure 1C). It is established that N-glycosylation proceeds in two steps: initial glycan attachment to the unfolded protein in the ER (core glycosylation), and further processing of the folded protein in the Golgi complex (complex glycosylation). At the cellular level, N-glycosylation is involved in protein folding and quality control (for a recent review, see Moremen et al., 2012). In accordance, DD1 ClC-5 mutants exhibiting defective N-glycosylation are retained in the ER and degraded at a faster rate (Grand et al., 2011). ClC-5 G261E matched this phenotype; it was arrested intracellularly in interconnected intracellular membranes (Figure 1E). Colocalization with ER tracker Red (Supplementary Figure 1, Pearson’s coefficient 0.93 ± 0.03, *n* = 5) confirmed that the mutant is retained in the ER. In contrast, ClC-5 WT was localized in the plasma membrane and on intracellular vesicles in HEK293T cells (Figures 1D,E).

### Barttin Regulates the Cellular Distribution and Ionic Transport of ClC-5

In the next step, we coexpressed and subjected to confocal imaging barttin and ClC-5 in HEK293T cells. The images revealed that the membrane abundance of ClC-5 in cells with high barttin expression is reduced. However, there was a significant cell-to-cell variation in the distribution of both proteins in co-transfected cells (Figure 1F). In some of the cells, both barttin and ClC-5 also stained the nuclear envelope or the perinuclear space. In agreement with the established ER arrest of the mutant, barttin coexpression did not alter the localization of ClC-5 G261E (Figure 1G). As an additional test, we performed analogous experiments using the Cercopithecus aethiops kidney cell line (COS-1). Confocal imaging showed that in these cells barttin has similar effects as observed in HEK293T cells (Supplementary Figure 2).

To estimate the plasma membrane abundance of ClC-5, we used limited digestion with the pancreas endoprotease α-chymotrypsin. A brief application of the enzyme to intact cells leads to the specific cleavage of surface-exposed protein chains. Quantifying the percentage of cleaved proteins can be used to determine the cell surface expression of a protein of interest with high precision (Hall and Soderling, 1997; Prasad et al., 2010). In our experiments, we expressed mCerulean-tagged ClC-5 either with or without mCherry-tagged barttin and quantified the ratio between the fraction of digested and intact proteins using SDS-PAGE and fluorescence densitometry. The analysis showed that the percentage of ClC-5 WT digested by chymotrypsin is reduced when barttin is co-expressed (Figures 1H,I). These results confirmed that barttin co-expression reduces the surface expression of ClC-5 in HEK293T cells. Combined, the reduced surface membrane abundance and complex N-glycosylation suggest that in HEK293T cells barttin coexpression prevents the exit of ClC-5 from the ER and its transport to the Golgi apparatus. In agreement with this hypothesis, brefeldin A, a known inhibitor of the flow from the ER to the Golgi (Sciaky et al., 1997), altered the subcellular distribution of ClC-5 in a manner that resembled the effects of barttin coexpression (Supplementary Figure 3). Because of its intracellular localization, mutant G261E ClC-5 was not digested (Figures 1H,I). Finally, we estimated the surface abundance of barttin and found it to be independent on the expressed ClC-5 construct (Figures 1H,J).

**FIGURE 2:**
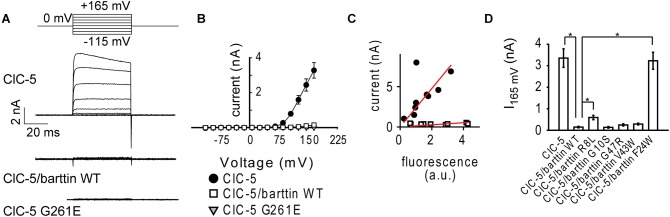
Electrophysiology measurements of ClC-5 ion transport in non-polarizing HEK293T cells. **(A)** Representative whole-cell patch–clamp current recordings of HEK293T cells expressing ClC-5 WT, ClC-5 G261E, or ClC-5 WT together with barttin WT. The current-voltage dependence of ClC-5 transport is depicted in panel **(B)**. **(C)** Correlation between ClC-5 steady-state current amplitude at +145 mV and whole-cell fluorescence for cells expressing ClC-5 WT or ClC-5 WT together with barttin WT. **(D)** Mean steady-state current amplitudes of ClC-5 expressed alone or together with WT or various mutants of barttin (6 < *n* < 25; The symbol “^∗^” indicates statistically significant differences with *p* < 0.05).

To test the functional effects of mutation G261E, we used whole cell patch clamp and measured ClC-5 currents in HEK293T cells transfected with mutant or WT ClC-5. The expression of the Dent disease mutant ClC-5 G261E did not result in detectable ionic currents (Figures 2A,B). These findings are in harmony with the predominantly intracellular localization of the mutant. Similarly, ionic transport mediated by ClC-5 WT was reduced when the transporter was coexpressed with barttin (Figures 2A,B). To test whether ion transport reduction is caused by reduced expression of ClC-5, we measured simultaneously ClC-5 current and fluorescence intensity of single cells transfected with Cerulean-tagged ClC-5. The fluorescence intensity in these experiments reports on the total amount of ClC-5 proteins expressed in a single cell. In contrast, the ionic current amplitude is proportional to the number of ClC-5 proteins residing in the plasma membrane. Independent on the presence of barttin, cells with higher ClC-5 expression (higher fluorescence intensity in the “cerulean” channel) exhibited higher electrogenic transport (Figure 2C). However, the slope of the data set was much smaller when barttin was coexpressed. Therefore, expression of the same number of ClC-5 proteins results in lower ionic current in the presence of barttin.

In the next step, we selected a number of barttin mutants and tested their effect on ClC-5 ionic transport. The first mutant was barttin F24W, a single amino acid exchange that dramatically impairs barttin protein stability (Wojciechowski et al., 2015). As expected, the coexpression of this mutant did not reduce ionic transport by ClC-5 WT (Figure 2D). We also tested three pathogenic BS4a mutations associated with BS – barttin R8L, G10S, and G47R (Birkenhäger et al., 2001; García-Nieto et al., 2006). In addition, we tested the effects of barttin V43W, an artificial mutation at the position of the polymorphism V43I discovered in patients with essential hypertension (Sile et al., 2007). All these barttin variants were capable of reducing ClC-5 transport. However, the extent of the reduction varied significantly between the mutants (Figure 2D). The differential regulation and the BS4a association of the mutants suggest that the barttin mediated regulation of ClC-5 transport is physiologically relevant.

### Effects of Barttin on the Processing of ClC-5

**FIGURE 3:**
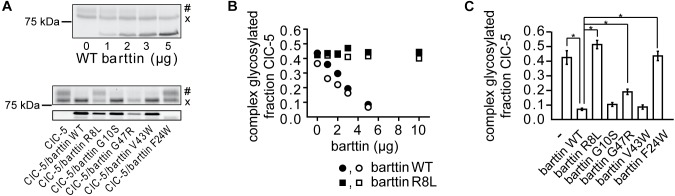
Effects of barttin on the glycosylation of ClC-5 in non-polarized HEK293T cells. **(A)** Grayscale representation of a fluorescent SDS-PAGE gel of ClC-5-mVenus expressed in HEK293T cells together with variable amounts of plasmids coding for WT barttin (upper panel) or together with 5 μg plasmid DNA encoding WT or various barttin mutants (lower panel). When expressed alone, a significant percentage of ClC-5 is complex glycosylated (#); the relative amount of the non-complex-glycosylated form (x) of ClC-5 is increased in the presence of barttin. **(B)** Quantitative analysis of four independent experiments testing the concentration-dependent effects of barttin WT (*n* = 2) and barttin R8L (*n* = 2) on the N-linked glycosylation of ClC-5 obtained as shown in panel **(A)**. The integrated intensity of the heavier ClC-5 band was normalized to the sum of the intensities of both bands in each lane. **(C)** Summarized effects of the barttin mutants shown in panel **(A)** on the complex glycosylation of ClC-5 (*n* = 6). The analysis was performed as in panel **(B)**; The symbol “^∗^” indicates statistically significant differences with *p* < 0.05.

The altered cellular localization of ClC-5 (Figure 1) suggests that barttin might affect the posttranslational modification and the processing of ClC-5. To test this hypothesis, we quantified the N-glycosylation of the transporter. SDS-PAGE analysis demonstrated that barttin co-expression impairs the complex glycosylation of ClC-5 in a concentration-dependent manner (Figures 3A,B). These findings explain the significant variation in the ClC-5 localization observed in the confocal images of cells coexpressing barttin (Figure 1). The previously tested barttin mutants (Figure 2) also impaired the complex glycosylation of ClC-5 (Figures 3A,C). Surprisingly, coexpressing mutant barttin R8L did not alter the glycosylation pattern of ClC-5. Gel band densitometry showed that the expression of barttin R8L is reduced by half compared to barttin WT, so we tested whether reduced expression of the mutant might be responsible for the lack of detectable effects. Increasing the amount of co-expressed barttin R8L to the level of barttin WT also did not affect the glycosylation of ClC-5 (Figure 3B). Thus, poor expression of mutant barttin R8L does not explain the lack of effects on the posttranslational processing of ClC-5.

We tested additionally how the coexpression of barttin affects the expression of ClC-5 (Supplementary Figure 4A). The analysis showed that the relative ClC-5 expression (normalized to the expression of barttin) is nearly the same for all investigated barttin mutants, except for barttin F24L. Therefore, the differentials effects on ClC-5 glycosylation are probably not linked to unspecific overexpression effects. We considered also the possibility that overexpression of barttin might non-specifically increase the degradation of ClC-5. However, the unaltered density of the ClC-5 lower density bands suggests otherwise (Supplementary Figures 4B–D). The differential effects of the BS4a mutants R8L and barttin G47R demonstrate, therefore, that disease-causing mutations are capable of specifically and differentially altering the effects of barttin on ClC-5 processing.

**FIGURE 4:**
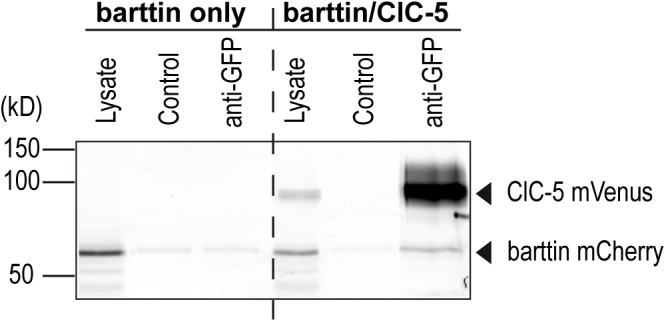
Co-immunoprecipitation of barttin and ClC-5. Grayscale presentation of a fluorescence scan of SDS-PAGE gel (*n* = 5). HEK293T cells were transfected with plasmids encoding ClC-5-mVenus and barttin-mCherry. An anti-GFP antibody was used to purify ClC-5-mVenus from cleared lysates using Protein-G-agarose beads. Barttin-mCherry could be co-purified with ClC-5-mVenus as seen in the anti-GFP lane indicating an association of ClC-5 and barttin. The specificity of the antibody is demonstrated by the lack of a signal in the anti-GFP treated lysate from cells expressing barttin-mCherry alone. Control lanes without antibody treatment show weaker barttin staining representing unspecific binding of barttin mCherry to the agarose beads.

### Evidence for Direct Interaction Between Barttin and ClC-5

In the next step, we performed co-immunoprecipitation experiments to test whether ClC-5 can directly bind to ClC-5. Using an anti-GFP antibody, barttin was co-purified with ClC-5-mVenus from lysates of transfected HEK293T cells (Figure 4). As in the case of barttin and ClC-K channels (Stölting et al., 2015), the co-purification indicates a potential ClC-5/barttin complex formation.

## Discussion

Our investigations provide experimental evidence that barttin can regulate the trafficking and processing of the Cl/H exchanger ClC-5. The existence of such a mechanism suggests that ClC-5 transport regulation might be part of the physiological repertoire of barttin. It could be argued that the observed effects are unspecific and result from the excessive overexpression of barttin. The most compelling argument against this possibility is provided by the BS4a mutant barttin R8L that does not affect the cellular localization and N-glycosylation of ClC-5 (Figures 1–3). Additional support is provided by the variable effects exerted by the rest of the investigated BS4a barttin mutants on ClC-5 trafficking, processing, and transport function. An argument against a non-specific ER stress induced by the overexpression of barttin is provided by the observation that neither ClC-5 protein degradation is increased, nor the expression of ClC-5 is excessively reduced in cotransfected cells (Supplementary Figure 4; Wagner et al., 2006). The pathogenic nature of the investigated barttin mutants implies, further, that altered ClC-5 transport might contribute to the phenotypic heterogeneity of BS4a. Interestingly, mutant barttin R8L suppressed ClC-5 transport despite lacking effects on glycosylation. It has been previously shown that this particular mutant (like two other pathogenic mutants) abolishes ClC-K channel ionic transport without preventing the insertion of the channel in the membrane (Janssen et al., 2009). The behavior has been explained by altered ClC-K channel gating. The resemblance to the effects observed by us suggests that barttin might also regulate the gating of ClC-5.

Our findings differ from results of previous investigations (Estévez et al., 2001; Waldegger et al., 2002) that did not detect interactions between barttin and ClC-5. For the following reasons, we believe that the expression of barttin in these earlier investigations might have been too low. Firstly, we could show that the effects of barttin are concentration-dependent (Figure 3). Secondly, a slight ClC-5 transport reduction in the presence of barttin (approximately 10%) was reported also in the aforementioned studies (Estévez et al., 2001; Waldegger et al., 2002). Finally, it was not known until recently that mammalian CLC anion/proton exchangers transport at much slower unitary rates compared with the single channel amplitudes of ClC-K channels (Scholl et al., 2006; Zdebik et al., 2008; Grieschat and Alekov, 2012). Therefore, macroscopic currents with similar amplitudes, as reported previously (Estévez et al., 2001; Waldegger et al., 2002), correspond to dramatically lower numbers of expressed ClC-K channels compared to the number of expressed ClC-5 transporters.

To our knowledge, we report here for the first time results from functional characterizations of the ClC-5 mutant G261E that has been associated with Bartter-like symptoms. The investigations showed that the mutant is retained in the ER due to improper processing and trafficking. Such a phenotype is not unusual and is often observed also in DD1 ClC-5 mutants not associated with Bartter like phenotype (see for example Grand et al., 2011). Therefore, the phenotype cannot explain the occurrence of Bartter-like symptoms in the affected patient, supporting the notion that additional proteins are involved in the disease pathophysiology. A speculative theory based on our findings could involve a competition between ClC-5 and ClC-K channels in the ER. Specifically, the increased abundance of ClC-5 G261E in the ER could reduce the ClC-K trafficking to the surface membrane and produce the atypical symptoms observed in the affected patients. To our knowledge, the co-occurrence of barttin mutations or polymorphisms in DD1 has not been tested. Neither has been investigated in detail how altered ClC-5 transport affects the function of the thick ascending limbs of the Henle’s loop or the kidney collecting duct. In this context, our results highlight the need for further research targeting this issue and demonstrate that the range of clinical and physiological investigations of Dent disease 1 should be extended.

Because of the prominent effects of ClC-5 in the PT, the physiological role of this transporter in the TAL and ICCD seems to be underrated. However, expression of ClC-5 in these segments is well documented (Günther et al., 1998; Luyckx et al., 1998; Devuyst, 1999; Sakamoto et al., 1999; Nanami et al., 2015; Ogawa et al., 2017; see Figure 1). Several studies demonstrate that this expression has a physiological impact. Specifically, an increased ClC-5 expression has been found in mouse medullary thick ascending limb cells under hypertonic conditions (Pham et al., 2004). Moreover, pathophysiological ablation of ClC-5 in human patients resulted in abnormal depletion of H-ATPases from the apical pole of α-type intercalated cells (Moulin et al., 2003). Last but not least, enhanced calcium crystal agglomeration in collecting duct epithelial cells has been observed upon disruption of ClC-5 (Carr et al., 2006). Remarkably, a physiological role for ClC-5 in podocytes has been also proposed and a mutation in ClC-5 has been linked to the occurrence of atypical focal segmental glomerulosclerosis (Solanki et al., 2018). Therefore, kidney ClC-5 transport appears to be essential not only for the PT function but also for the proper function of other nephron segments.

Bartter-like symptoms are uncommon for DD1. However, two recent studies including a large pool of affected individuals report a decline of plasma potassium concentration with age in approximately 40% of DD1 patients while bicarbonatemia remains in the normal range (Mansour-Hendili et al., 2015; Blanchard et al., 2016). These observations suggest that mild hypokalemia is actually typical for DD1. Therefore, it is tempting to speculate that the barttin-dependent regulation of ClC-5 trafficking and processing is altered not only by mutation G261E but also by other DD1 mutations and contributes to hypokalemia progression in the affected patients. In contrast, DD1 mutants like the here investigated G261E ClC-5 might impair additional renal regulatory cascades and increase the risk of metabolic alkalosis. The novel regulatory mechanism described here suggests a role for barttin as traffic controller regulating the processing and intracellular targeting not only of ClC-K channels but also of ClC-5 and probably of other CLC’s expressed in the kidney.

## Author Contributions

DW, EK, LY, CF, and AA contributed to the design of the work. DW, EK, LY, HT, GS, and AA contributed to acquisition, analysis, and interpretation of data. AA drafted the manuscript. DW, EK, LY, CF, GS, and AA revised the paper critically for important intellectual content.

## Conflict of Interest Statement

The authors declare that the research was conducted in the absence of any commercial or financial relationships that could be construed as a potential conflict of interest.
