# Supplementary material for: Barttin Regulates the Subcellular Localization and Posttranslational Modification of Human Cl-/H+ Antiporter ClC-5
Source: Front Physiol. 2018 Oct 23;9:1490. doi: 10.3389/fphys.2018.01490 (PMC6206076; doi:10.3389/fphys.2018.01490)
Supplement: Supplementary file 1 [file Data_Sheet_1.pdf]

Supplementary Information

Barttin Regulates the Subcellular Localization and Posttranslational Modification of Human Cl^-/H^+ Antiporter ClC-5

D. Wojciechowski[1#], E. Kovalchuk[1#], L. Yu[1], Hua Tan[2], Ch. Fahlke[2], G. Stölting[2], A. K. Alekov[1*]

[1]Institute for Neurophysiology, Hannover Medical School, D-30625 Hannover, Germany

[2]Institute of Complex Systems 4 (ICS-4) – Zelluläre Biophysik, Forschungszentrum Jülich, D-52425 Jülich, Germany

[#] contributed equally

*** Correspondence:**
A. K. Alekov
alexi.alekov@gmail.com

Keywords: ClC-5, barttin, CLC transport, kidney, Dent disease, Bartter syndrome, Golgi bypass

1 supplementary table and 4 supplementary figures

Supplementary Table 1

ClC-5 protein mutation	Mutation type	Reference	Atypical symptoms
R347*	nonsense	Besbas et al., 2005	hypokalaemic metabolic alkalosis; secondary hyper-reninaemic hyperaldosteronism
G261E	missense	Bogdanović et al., 2010	hypokalemic metabolic alkalosis; hyperreninemic hyperaldosteronism; growth hormone (GH) deficiency.
Y567*	nonsense	Okamoto et al., 2012	hypokalemic metabolic alkalosis, incerased levels of plasma renin activity and aldosterone
D727M_fs*3	frameshift	Sheffer-Babila et al., 2008	growth hormone deficiency

Supplementary Table 1. ClC-5 mutations associated with atypical normochloremic hypokalemic metabolic alkalosis, hyperaldosteronism, and/or growth hormone deficiency

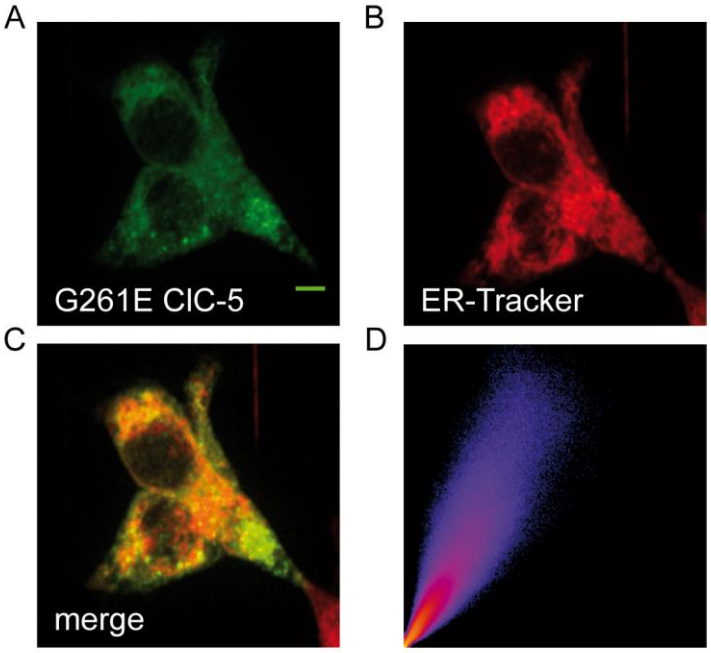

Supplementary Figure 1. Colocalization analysis of G261E ClC-5 YFP and ER-Tracker Red in transfected in HEK293T cells. Cells transfected with mutant G261E ClC-5 YFP were incubated with ER Tracker Red and confocalized in 3D (see Methods in the main text). **A)** Confocal image depicting the localization of G261E ClC-5 YFP in z-projection. **B)** Confocal image of the same cells depicting the distribution of ER Tracker Red in z-projection. **C)** Dual-color image constructed from merging images in A) and B). **D)** Cytofluorogram illustrating the pixel-based Pearson's colocalization of the cells depicted in A) and B).

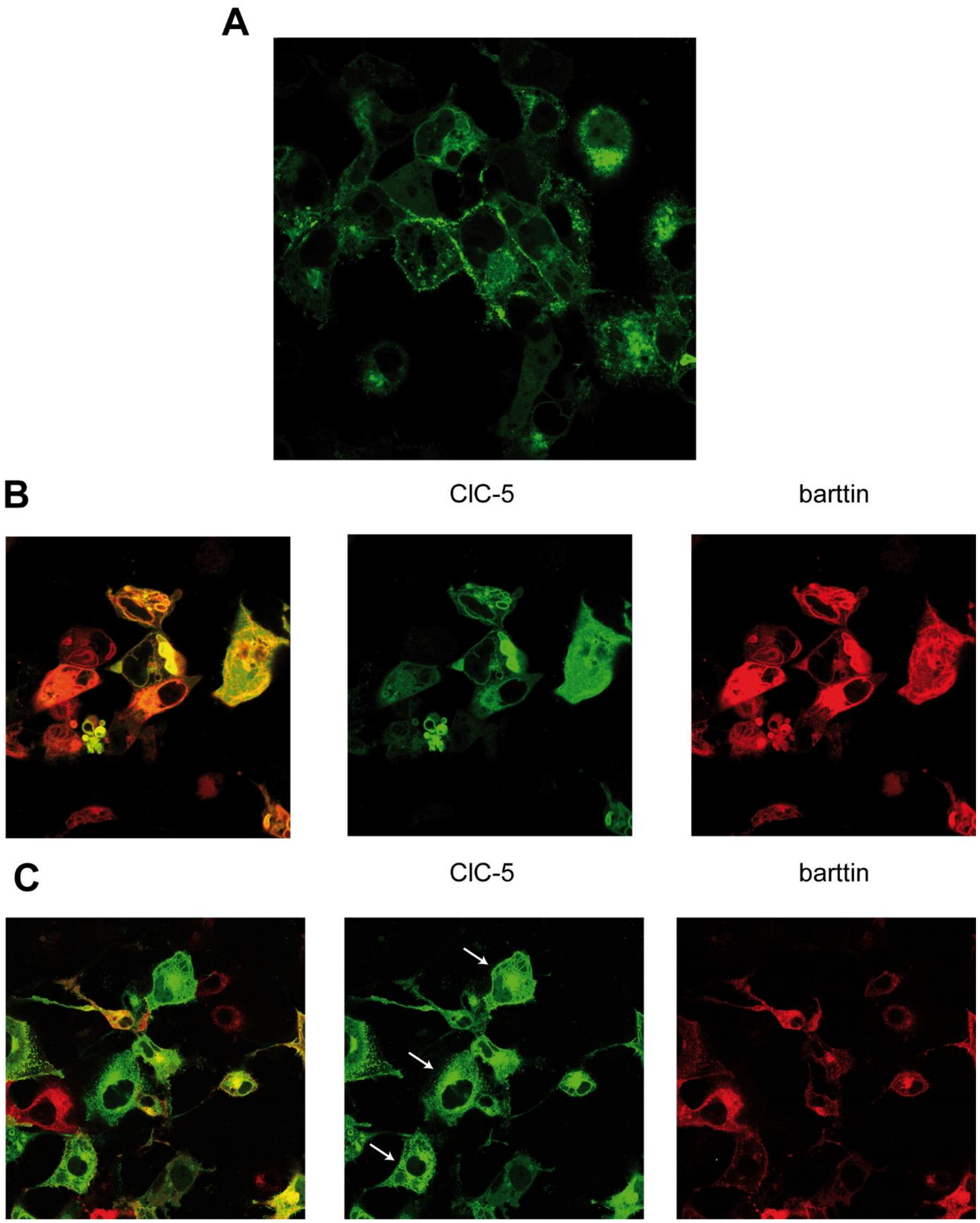

Supplementary Figure 2. Effects of barttin coexpression on ClC-5 investigated in COS-1 cells A) Representative confocal image of COS-1 cells expressing ClC-5 GFP **B)** Composite confocal image of COS-1 cells expressing 5 µg of ClC-5 GFP (green) and 1 µg barttin mCherry (red). The separate confocal channels are depicted in green (ClC-5 GFP) and red (barttin mCherry). **C)** Composite confocal image of COS-1 cells expressing 5 µg of ClC-5 GFP (green) and 0.5 µg barttin mCherry (red). The separate confocal channels are depicted in green (ClC-5 GFP) and red (barttin mCherry). Arrows indicate cells with low barttin expression.

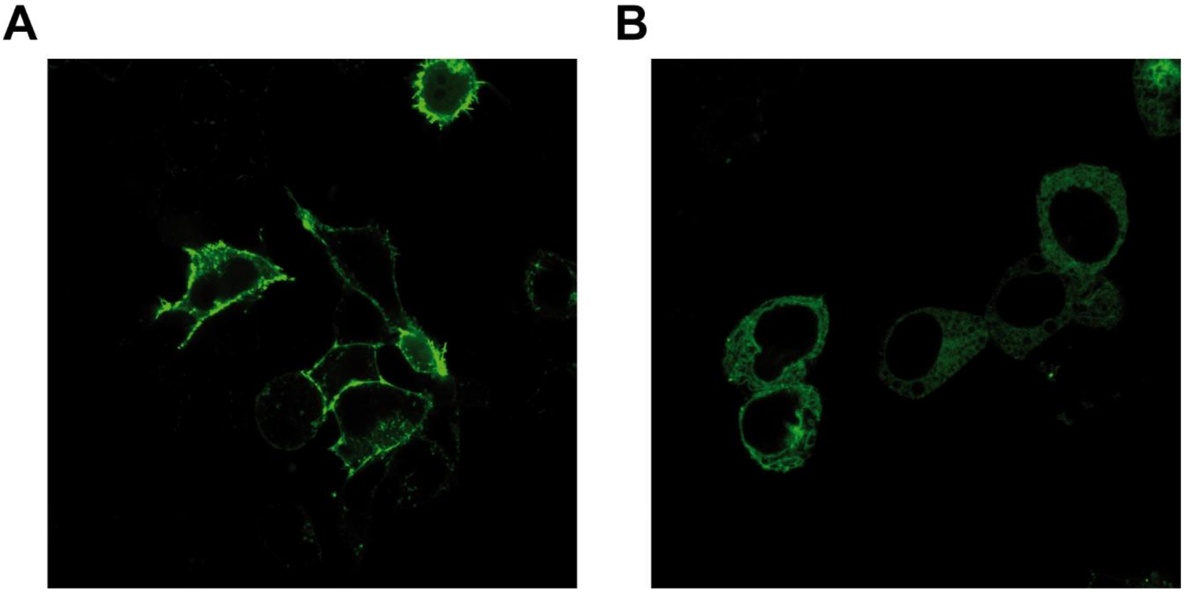

Supplementary Figure 3. Effects of blocking the ClC-5 ER-to-Golgi transport by brefeldin A
A) Representative confocal image of HEK293T cells expressing ClC-5 GFP **B)** confocal image of HEK293T cells expressing ClC-5 GFP that have been incubated for 16h before imaging with 5µg/ml brefeldin A.

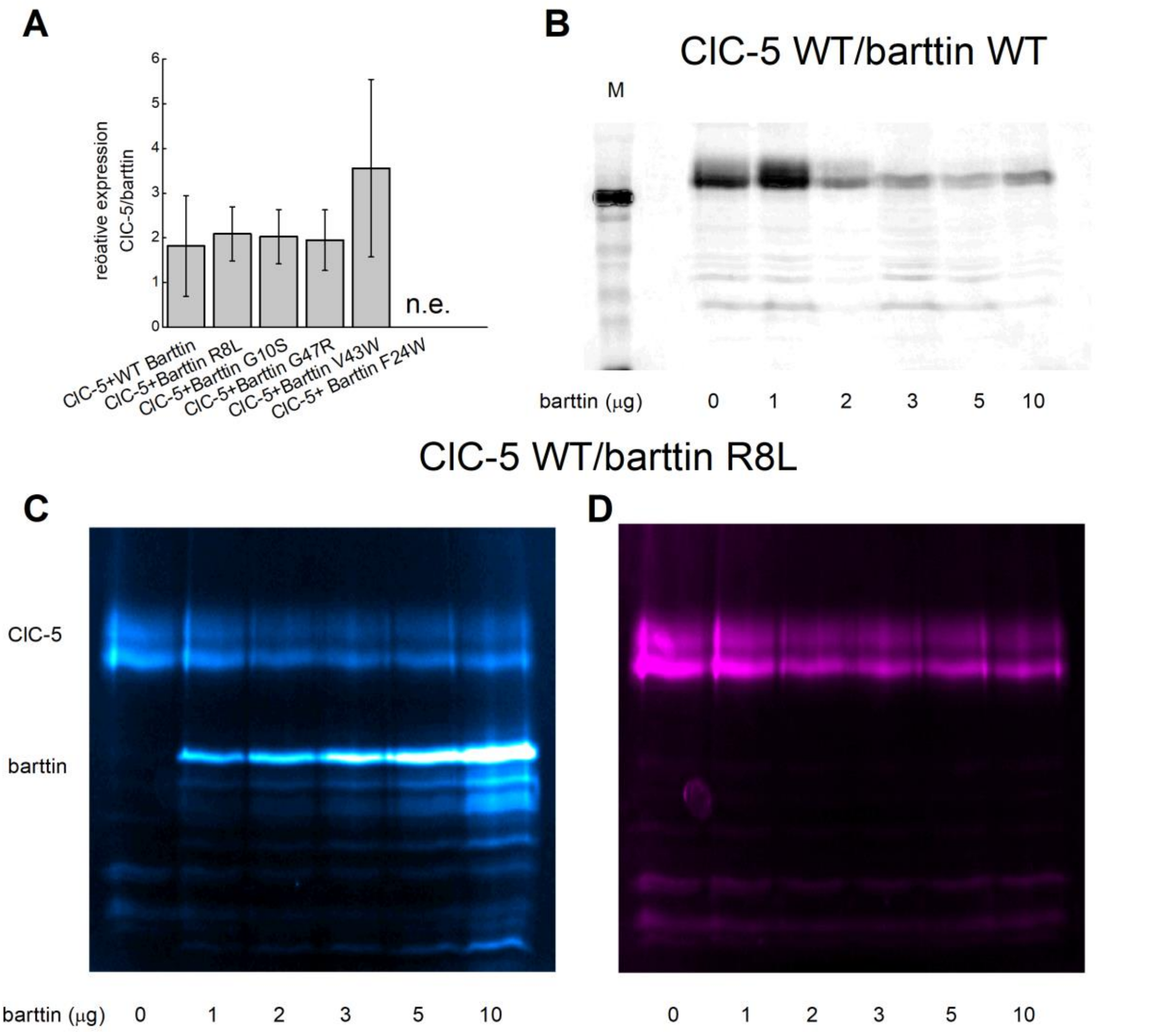

Supplementary Figure 4. Effects of barttin coexpression on the expression and stability of ClC-5
A) Analysis showing the relative expression of ClC-5 as normalized to the expression of barttin in the same experiment. Densitometry analysis was performed on the same datasets presented in Fig. 3C. Analysis was not possible for barttin F24W (n.e. for not evaluated) because of the severe degradation of the mutant and the unreliable detection of the smaller bands (a significant amount was lost because of the small sizes). **B)** Grayscale representation of a fluorescent SDS-PAGE gel of ClC-5-mVenus expressed in HEK293T cells together with variable amounts of plasmids coding for WT barttin. **C)** and **D)** False color representation of a fluorescent SDS-PAGE gel of ClC-5-mVenus expressed in HEK293T cells together with variable amounts of plasmids coding for barttin R8L. Two channels were recorded to better show barttin (cyan) and ClC-5 (magenta). Please note the gradual increase of barttin expression observed upon increasing the amount of transfected DNA.
